# Host Range and Coding Potential of Eukaryotic Giant Viruses

**DOI:** 10.3390/v12111337

**Published:** 2020-11-21

**Authors:** Tsu-Wang Sun, Chia-Ling Yang, Tzu-Tong Kao, Tzu-Haw Wang, Ming-Wei Lai, Chuan Ku

**Affiliations:** 1 Institute of Plant and Microbial Biology, Academia Sinica, Taipei 11529, Taiwan; r09b48007@ntu.edu.tw (T.-W.S.); as0190267@gate.sinica.edu.tw (C.-L.Y.); tzutongkao@gate.sinica.edu.tw (T.-T.K.); tzuhawwang@gate.sinica.edu.tw (T.-H.W.); mwlai@gate.sinica.edu.tw (M.-W.L.); 2 Genome and Systems Biology Degree Program, National Taiwan University and Academia Sinica, Taipei 10617, Taiwan

**Keywords:** Nucleo-Cytoplasmic Large DNA Viruses (NCLDVs), algae, protists, cophylogeny, host switch, auxiliary genes, virus-encoded metabolism, gene repertoire, genome evolution, lateral gene transfers

## Abstract

Giant viruses are a group of eukaryotic double-stranded DNA viruses with large virion and genome size that challenged the traditional view of virus. Newly isolated strains and sequenced genomes in the last two decades have substantially advanced our knowledge of their host diversity, gene functions, and evolutionary history. Giant viruses are now known to infect hosts from all major supergroups in the eukaryotic tree of life, which predominantly comprises microbial organisms. The seven well-recognized viral clades (taxonomic families) have drastically different host range. *Mimiviridae* and *Phycodnaviridae*, both with notable intrafamilial genome variation and high abundance in environmental samples, have members that infect the most diverse eukaryotic lineages. Laboratory experiments and comparative genomics have shed light on the unprecedented functional potential of giant viruses, encoding proteins for genetic information flow, energy metabolism, synthesis of biomolecules, membrane transport, and sensing that allow for sophisticated control of intracellular conditions and cell-environment interactions. Evolutionary genomics can illuminate how current and past hosts shape viral gene repertoires, although it becomes more obscure with divergent sequences and deep phylogenies. Continued works to characterize giant viruses from marine and other environments will further contribute to our understanding of their host range, coding potential, and virus-host coevolution.

## 1. Introduction

The Nucleo-Cytoplasmic Large DNA Viruses (NCLDVs) are a group of double-stranded DNA viruses of eukaryotes that was established two decades ago [1]. Analyses of few widely distributed core genes suggest a monophyletic origin of NCLDVs [1,2], which have been formally named *Nucleocytoviricota* within the virus kingdom *Bamfordvirae* (realm *Varidnaviria*) by the ICTV [3,4]. For their extraordinary genome size (up to 2.8 Mb [5]) and virion size (up to 2.5 μm [6]) among all viruses, NCLDVs are commonly known as giant viruses [7,8,9,10,11], which reflects their distinction from traditionally defined viruses [12]. Although arbitrary thresholds can be applied to draw a line between *large* and *giant* viruses, it is now clear that NCLDV genomes larger than a certain size (e.g., 500 [13] or 300 [14] kb) have multiple evolutionary origins and that their size distribution forms a continuum with considerable variation both within and across families [13,15]. Still, little is known about the mechanisms that underpin the evolution and maintenance of giant virus genome diversity.

Recent advances in the biology of giant viruses have brought to the fore their expanded host range and coding potential, as shown in numerous studies based on isolation and cultivation, genomic and functional characterization, or environmental metagenomics. Giant viruses have been found in a myriad of eukaryotes previously unknown as hosts, gradually filling the gaps of giant virus hosts in the eukaryotic tree of life. Newly sequenced genomes often contain genes that have rarely or never been found in viruses, while the majority of genes in giant viruses do not even have homolog matches in sequence databases. The virus-encoded genes that are otherwise characteristic of cellular organisms could play crucial roles in manipulating the metabolism of infected cells, converting them into virocells [16,17]. By considering NCLDVs—giant viruses—as a whole, here we briefly summarize and highlight recent findings that have revolutionized our view of their host range and coding potential, with particular emphasis on the evolutionary implications for giant viral genomes.

## 2. Giant Viruses Infect Every Major Eukaryotic Lineage

### 2.1. The Founding Members of NCLDVs

**Figure 1:**
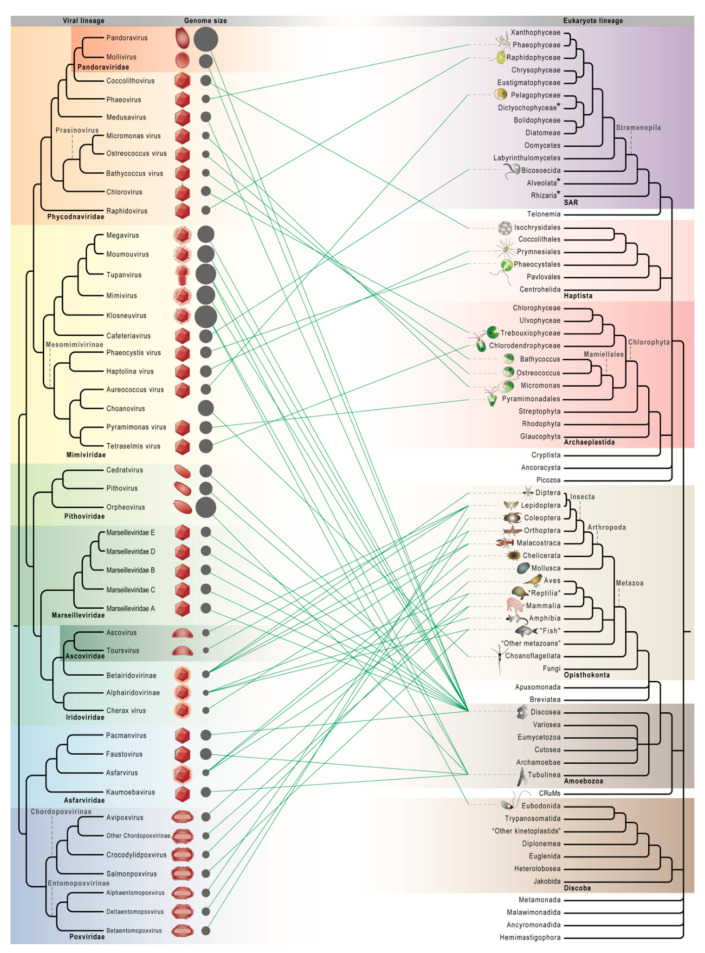
Cophylogenetic relationships between giant viruses and eukaryotes. Virus-host connections are mapped onto reference trees summarizing giant virus phylogenies based on up to 10 putatively vertically inherited core genes [13,14,37,42,43] and phylogenetic relationships across the eukaryotic tree of life [27,44,45,46,47,48,49,50]. The seven commonly delineated giant virus clades (seven families with two nested families) are divided into subfamilial lineages, each of which comprises one or more viral strains with known hosts (Table S1), with a schematic diagram of virion morphology, if known, and the average genome size across strains (proportional to the circle area; Table S1) shown. Names in double quotes correspond to non-monophyletic groupings. * Giant viruses are also known to infect the SAR lineages dictyochophytes (Stramenopila) [51,52], dinoflagellates (Alveolata) [53], and chlorarachniophytes (Rhizaria) [52], but their phylogenetic positions in the virus reference tree are less certain.

Our current knowledge of the host range of NCLDVs (Figure 1 and Table S1) is dramatically different from when NCLDVs were first recognized through comparative genomics and comprised by only four pre-existing families [1]: three families of animal (vertebrate and arthropod) viruses—*Poxviridae* (e.g., variola and vaccinia viruses), *Asfarviridae* (only African swine fever virus [ASFV]), and *Iridoviridae* (vertebrate and insect viruses, incl. *Ascoviridae* [18])—and the alga-infecting *Phycodnaviridae*, including chlorovirus of *Chlorella* (Trebouxiophyceae, Chlorophyta) and phaeovirus of the multicellular brown alga *Ectocarpus* (Phaeophyceae, Stramenopila). These first known hosts of NCLDVs were clearly biased toward human, livestock, and other multicellular organisms. In fact, only a few unicellular hosts of giant viruses had been reported before the establishment of NCLDV in 2001. Unlike the better studied chlorovirus [19], genome sequences of marine viruses infecting the green microalga *Micromonas* (Mamiellales, Chlorophyta) [20], the bloom-forming coccolithophore *Emiliania* (Isochrysidales, Haptista) [21], the heterotrophic flagellate *Cafeteria* (Bicosoecida, Stramenopila; originally misidentified as *Bodo*) [22], and the harmful bloom-forming alga *Heterosigma* (Raphidophyceae, Stramenopila) [23] became available only after 2001 [9,24,25,26], which eventually supported their grouping with other NCLDVs.

### 2.2. The Age of Discovery for Giant Viruses Infecting Microbial Eukaryotes

With the advancement in genome sequencing and environmental microbiology, NCLDVs have been expanding substantially in terms of known host diversity. A major shift to discovering giant viruses in microbial eukaryotes or protists, which comprise the vast majority of eukaryotic lineages [27], began with the recognition of mimivirus, a bacterium-like pathogen in *Acanthamoeba* (Discosea, Amoebozoa) and the first giant virus with an Mb-sized genome [8]. As the first non-photosynthetic protist host of giant viruses, *Acanthamoeba* (ubiquitous free-living amoebae and opportunistic human pathogens [28]) had since then become the model organisms for isolating giant viruses from a variety of environmental samples, leading to the discovery of marseillevirus [29], megavirus [30], moumouvirus [31], pandoravirus [5], pithovirus [32], mollivirus [33], cedratvirus [34], pacmanvirus [35], tupanvirus [36], and medusavirus [37]. A distantly related amoebal lineage, *Vermamoeba* (Tubulinea, Amoebozoa), has been used since 2015 as an alternative host system for viruses that cannot replicate in *Acanthamoeba*, including faustovirus [38], kaumoebavirus [39], orpheovirus [40], and yasminevirus (a klosneuvirus) [41]. Giant viruses isolated with this approach have given rise to the proposal of four new families—*Mimiviridae*, *Marseilleviridae*, *Pandoraviridae*, and *Pithoviridae*—and expanded some pre-existing families (Figure 1). These commonly recognized NCLDV families vary greatly in their virion morphology, genome characteristics, and replication cycle (Figure 1 and Table 1).

Giant viruses have also been found in other diverse aquatic microbial eukaryotes, greatly broadening their known host diversity. These include heterotrophic flagellates *Bodo* (Kinetoplastida, Discoba; infected by a klosneuvirus) [66] and *Bicosta* (Choanoflagellata, Opisthokonta) [14] and marine microalgae *Haptolina* (previously Chrysochromulina) (Prymnesiales, Haptista) [67], *Pyramimonas* (Pyramimonadales, Chlorophyta) [67], *Phaeocystis globosa* (Phaeocystales, Haptista) [68], *Aureococcus* (Pelagophyceae, Stramenopila) [69], *Florenciella* and *Rhizochromulina* (Dictyochophyceae, Stramenopila) [51,52], and Chlorarachniophyceae (Cercozoa, Rhizaria) [52]. As alternatives to isolation and cultivation, single-virion genomics [70], single-cell genomics [14], and metagenomics [14,43,71] are especially useful when the host eukaryotes are unknown or cannot be purified and cultured. These methods not only generate sequences of individual viruses but can also infer their putative hosts, such as *Bicosta* of choanovirus [14] or cercozoans (Rhizaria) of wastewater klosneuviruses [71].

### 2.3. Increasingly Non-Algal Phycodnaviridae and Increasingly Non-Amoebal Mimiviridae

Genomic information from newly reported viruses has often challenged family delineations of NCLDVs, in particular Phycodnaviridae and Mimiviridae, the two families with the most diverse host and genome size range (Figure 1). *Phycodnaviridae*, which literally means algal DNA viruses, originally encompassed only chlorovirus, and it was proposed that this family should include phaeovirus and *Micromonas* virus based on some common properties [7] despite the lack genome sequences at that time. These two viral lineages, as well as coccolithovirus, *Heterosigma* raphidovirus, and viruses of *Ostreococcus* and *Bathycoccus* (in the same order Mamiellales as *Micromonas*), turned out to be indeed closely related at the genomic level. By contrast, the story became more complicated for the other alga-infecting giant viruses. *Haptolina*, *Pyramimonas* and *Phaeocystis* viruses were suggested to be part of *Phycodnaviridae* [67,68], and so was *Aureococcus* virus despite some apparently contradictory molecular evidence [69]. It was only until phylogenetic and genomic analyses with mimivirids were conducted that it became clear that these viruses, along with the metagenomically discovered Organic Lake Phycodnaviruses (OLPVs) [72], more recently reported virus of *Tetraselmis* (Chlorodendrophyceae, Chlorophyta) [73], and choanovirus, are more closely related to mimivirids (incl. cafeteriavirus) [72,73]. It was further proposed that these viruses should form part of an extended *Mimiviridae* [11,13,42,74,75] or even the subfamily *Mesomimivirinae* within *Mimiviridae* [42,43,76] (Figure 1). *Mesomimivirinae* is certainly expanding as most newly reported alga-infecting viruses join this clade instead of *Phycodnaviridae*, such as the viruses from *Florenciella*, *Rhizochromulina*, a chlorarachniophyte, and *Prymnesium kappa* [51,52,77] (not shown in Figure 1). These, plus the viruses of heterotrophic flagellates *Cafeteria*, *Bodo*, and *Bicosta*, all transform *Mimiviridae* into a more non-amoebal virus family.

On the other hand, amoeba-infecting viruses have “invaded” other families. In addition to *Mimiviridae* and the two purely amoebal families, *Marseilleviridae* and *Pithoviridae*, viruses that infect *Acanthamoeba* or *Vermamoeba* have often been placed within the two NCLDV founding families *Asfarviridae* and *Phycodnaviridae* (Figure 1). *Asfarviridae*, with its name derived from ASFVs—notorious viruses that kill pigs and boars, can be transmitted through tick (Chelicerata, Metazoa) vectors, and have close relatives infecting abalones (Mollusca, Metazoa) [64,78] (Table S1)—has recently been joined by related amoebal viruses with genomes smaller than 500 kb [13,35,38,39] and possibly also a marine algal virus infecting *Heterocapsa* (Dinoflagellata, Alveolata) [53]. *Phycodnaviridae* has lost some alga-infecting members, but at the same time, it has been joined by amoebal viruses characterized by the largest known genomes—pandoravirus. Phylogenetic analyses based on core genes have strikingly and consistently nested *Pandoraviridae* (here incl. mollivirus) within *Phycodnaviridae* and as sister to the alga-infecting coccolithovirus with a much smaller genome [13,14] (Figure 1). Besides, the more recently discovered medusavirus could also be related to this subclade of *Phycodnaviridae* based on the phylogenies of major capsid protein [37] or 12 core genes [79] (Figure 1). With the extended host range, giant virus families that are cladistically defined based on core gene phylogenies are obviously not confined to their prototypic host, be it amoebal, algal, or swine.

### 2.4. Undiscovered Virus–Host Relationships

To date, giant virus infection has been reported from each of the most taxon-rich and well established lineages (supergroups) of eukaryotes [27,80], including Opisthokonta, Archaeplastida, SAR (incl. each of the three subgroups Stramenopila, Alveolata, and Rhizaria), Haptista, Amoebozoa, and Discoba (part of the now non-monophyletic “Excavata” that also includes Metamonada and Malawimonada) (Figure 1). Eukaryotes from which giant viruses were first isolated tend to be more relevant or observable to humans, whereas more recently discovered giant viruses are mostly from relatively understudied protist lineages of which research has been greatly accelerated by high-throughput genome sequencing. In addition to the known virus–host relationships (Figure 1 and Table S1), several lines of evidence are pointing to the immense diversity of undiscovered viruses and their hosts. Individual genomes assembled from metagenomic sequencing data (i.e., metagenome-assembled genomes [MAGs]) have led to the discoveries of OLPVs from Antarctica [72], 16 giant viruses from soil [43], hundreds of aquatic MAGs related to *Mimiviridae* and *Phycodnaviridae* [17], and over 2000 giant virus MAGs from across the globe [81], which greatly outnumber the currently described members in giant virus families. Genome sequences of eukaryotes have also hinted at putative (past) associations between giant viruses and Streptophyta, Cryptista, Fungi, and many other eukaryotic lineages where giant viruses have not been isolated [75]. However, despite similar evidence for some land plants [82], or embryophytes (Streptophyta), the complete lack of giant virus reported from any plant could indeed represent a small gap of giant virus host range on the tree of eukaryotes. This could be explained by the fact that plant viruses usually take advantage of the plasmodesmata aperture to spread systemically and encode movement proteins for intercellular transport through plasmodesmata [83], which is unlikely for the large size of giant virus particles or genomic DNA.

## 3. Variation and Evolution of Host Range 

At the level of individual virus, most giant viruses are known to infect only specific hosts. However, it is often uncertain whether the known hosts are the only and natural hosts. Because of the systematic isolation approach, many giant virus lineages are only known to infect *Acanthamoeba* or *Vermamoeba*, resulting in the pattern of multiple viruses connected to only one host (Figure 1). It could be that these widely occurring amoebae are indeed the natural, specific host of all those viruses, that they are simply more permissive lab hosts in which a wide range of viruses can be propagated, or that they are secondary hosts for those viruses with primary hosts and serve as the genomic melting pot [29] for various giant viruses. It should be noted that, with few exceptions such as tupanvirus [36], lab experiments have demonstrated that amoebal viruses can only replicate within *Vermamoeba* but not *Acanthamoeba* [38,39,40,41], or vice versa [84], suggesting there is still virus-host specificity between the two permissive hosts.

At the family level, giant virus families show wide variation in the extent of host range. *Poxviridae* and *Iridoviridae* (incl. *Ascoviridae*) infect only animals (Metazoa, Opisthokonta) or more specifically only vertebrates and arthropods. Within each of them exist subclades with narrower host range, e.g., vertebrates (Chordopoxvirinae and Alphairidovirinae) or arthropods (Entomopoxvirinae and Ascoviridae-Betairidovirinae). The more recently established *Marseilleviridae* and *Pithoviridae* also have narrow host range (Amoebozoa). On the contrary, *Phycodnaviridae* is associated with four eukaryotic supergroups and *Mimiviridae* with six in total. This host diversity cannot be attributed to their numbers of described viruses, which are dwarfed by that of *Poxviridae* or *Iridoviridae* (Table S1). Neither can it be explained by their intrafamilial phylogenetic divergence in terms of core genes [13,42,43], which is obviously higher than among the isolated members of Marseilleviridae but comparable to that of *Poxviridae* or *Iridoviridae*. The major difference between wide– and narrow–host-range viral families is probably the extent of genomic variation (Figure 1). This is evidently greater within Phycodnaviridae or Mimiviridae than within Poxviridae or Iridoviridae with generally small genomes, implying plasticity and variability in genome content could be key to conquering a wider range of hosts.

Insights into virus-host specificity have also been gained from studies on closely related viruses. A cross-infection network between coccolithovirus and *Emiliania huxleyi* strains showed a nested host-virus interaction pattern where more resistant hosts are only infected by viruses with broader host range, suggesting strong coevolution in host-virus system [85]. At a larger scale, phylogenetic correspondence has been observed between three genera of Mamiellales (“Prasinophyceae”, Chlorophyta) and their prasinoviruses [44]. Some discrepancy (i.e., imperfect cophylogeny and non-monophyly of *Ostreococcus* viruses) does exist between the trees of viral DNA polymerase and algal ribosomal RNA genes [44], but this can also be seen in strictly vertically inherited symbiont-host system [86] and can be due to incomplete lineage sorting, choice of genes, or taxon sampling. A later phylogenetic analysis with 22 genes from fewer strains of prasinoviruses resolved *Ostreococcus* viruses as monophyletic [87]. Overall, the cophylogenetic pattern indicates long-term coevolution between Mamiellales and prasinoviruses, with either cospeciation or host-switching events [44]. In contrast to host variation between closely related viral strains, processes of host change or expansion involving phylogenetically distant eukaryotes still remain largely unknown. 

## 4. Functional Potential of Virus-Encoded Proteins

Most predicted genes in giant viruses have unknown functions, and many of them have no homolog match in sequence databases at all [55]. In addition to near-universal core genes fundamental in virus replication cycle (e.g., DNA polymerase, primase-helicase, major capsid protein, genome packaging ATPase, transcription factor VLTF3 [13,88]), the minority of genes with functional predictions and cellular homologs often show unprecedented occurrence in the viral world (Table 2). The expanded genome size of giant viruses paves the way for harboring a large variable portion of the genome encoding auxiliary metabolic genes [16] (virus-encoded metabolism) and genes with other functions. They can allow for finer modulation of metabolism, gene expression, and behaviors in diverse hosts, converting them into virocells [16,17] during infection and playing a key role in the virus-host interaction and genome evolution (Table 2).

### 4.1. Information Storage and Flow 

Giant viruses exert control over different levels of genetic information in a cell. In addition to their own DNA polymerase and ligase for genome replication, some giant viruses encode DNA glycosylase involved in base excision repair pathways that could potentially remove damages to their large genomes [77,99]. Most NCLDVs also encode DNA-dependent RNA polymerase (DDRP) subunits (Table 1) with architectural modifications that confer them higher speed and processivity than the cellular homologs [54]. Interestingly, in *Phycodnaviridae* (as defined in Figure 1), these genes are only found in coccolithovirus [24], pandoravirus [5], and mollivirus [33], which have the largest genomes within the family (Figure 1). Except medusavirus [37], all those phycodnavirids without DDRP genes infect algae (Figure 1), which is in sharp contrast to the alga-infecting mimivirids (in subfamily *Mesomimivirinae*; Figure 1) that have the most complete complement of DDRP subunits among giant viruses [54]. Giant viruses also have various transcription factors involved in basic transcriptional regulation (initiation, elongation, and termination) and expressional control of viral kinetic classes [9,54,128,129]. Some unknown genes could further rewire the entire cellular transcriptomes, such as differential shut-down of nucleus- and organelle-encoded transcripts [129]. Presence of genes for translational control is a major hallmark of giant viruses. Except ribosomal proteins or RNA, a wide range of translation system components can be encoded, including tRNAs (Table 1), aminoacyl tRNA synthetases, and translation factors [13,71] (Table 2). There can be extensive variation in the repertoire of these translational machinery genes even among closely related viruses, for example, klosneuviruses (*Mimiviridae*) where *Bodo* virus has completely lost all its tRNAs while some others have nearly all the translation machinery genes found in giant viruses [13,66,71]. Whereas informational genes generally comprise the essential and core components in genomes of cellular organisms, the extreme variation among viruses with similar hosts or close phylogenetic relationships further demonstrates the plasticity and variability of giant virus genomes.

### 4.2. Energy Metabolism

The requirements for energy during genome replication, gene expression, and virus assembly make the control of energy metabolism a natural target of giant viruses. Such control can be transcriptional regulation of nucleus- and mitochondrion-encoded genes related to energy metabolism, as in coccolithovirus [129]. Recently reported genome sequences of environmental MAGs [17] or isolated viruses even encode their own genes related to glycolysis, tricarboxylic acid cycle, succinate dehydrogenase, β-oxidation, and photosynthesis [17,77,81,101]. Genes encoding enzymes in cellular fermentation, such as pyruvate formate-lyase, have been found in *Tetraselmis* virus infecting green algae [73], which have anaerobic energy metabolism in low-oxygen condition [130]. 

### 4.3. Synthesis of Biomolecules

Giant viruses encode various proteins participating in the synthesis of different virion components, with notable examples in carbohydrate, lipid, and nucleotide metabolism. Chloroviruses have plenty of carbohydrate metabolic genes for synthesis of hyaluronan, nucleotide sugars, glycans, and glycoproteins (e.g., capsid proteins glycosylated with distinct glycan structures) [89,90,91,131]. Coccolithovirus encodes unique host-derived genes for making virus-specific glycosphingolipids that not only constitute the virion membranes but induce host programmed cell death [16,24,114,115,132]. To meet the demand of nucleotides for synthesis of genomic DNA and RNA transcripts, nucleoside-diphosphate kinase and reductase are encoded by multiple giant viruses for nucleotide synthesis and conversion [24,66,104], which can be coupled with induction of host pentose phosphate pathway to enlarge the pool of available nucleotides [132,133].

### 4.4. Membrane Transport and Sensing

Giant viruses not only take control of information flow, energy metabolism, and biosynthesis but can also alter interactions between the cell and the environment through membrane proteins. A variety of such proteins, including rhodopsins, channels, and transporters, are encoded in the genomes of *Mesomimivirinae* and alga-infecting phycodnavirids. Type-1 rhodopsin genes are found in OLPVs, *Phaeocystis* virus, and Choanovirus, where they pump protons as a light-dependent energy transfer system [10,14]. Choanovirus additionally possess biosynthesis genes for the rhodopsin chromophore, retinal, which are absent in *Phaeocystis* virus but present in its host *Phaeocystis* [14]. A newly discovered type of rhodopsins—heliorhodpsins—is encoded in coccolithovirus genomes [134], which could play a role in light sensing during virus-host interactions. Light-gated anion-conducting channelrhodopsins have recently been found to be encoded in metagenomic contigs of *Mesomimivirinae* and Phycodnaviridae, probably transferred from Pyramimonadales green algae, and could be used to change the host’s swimming behavior in response to light [79]. Potassium channels are commonly encoded by algal viruses in Phycodnaviridae and *Mesomimivirinae* and in the chlorovirus-*Chlorella* system they cause membrane depolarization, decrease turgor pressure, and promote viral DNA ejection [51,116,117,118,119]. The potential function in other algal viruses could be to make the intracellular environment more favorable to virus-encoded proteins [51], which might also be achieved by the calcium transporting ATPase encoded in chlorovirus [124]. Furthermore, nutrient transporters, including ammonium [87] and sodium/phosphate [121,122] transporters, are commonly encoded in the genomes of algal giant viruses. Some of the aforementioned membrane proteins are brought into the virus-host system by viruses, while some are encoded in both the viral and host genomes. In the latter case, it is often found that the viral and host homologs have different activities or substrate affinities. For example, the ammonium transporter unique to one *Ostreococcus* virus shows higher uptake rate than the host counterpart at lower substrate concentrations and can potentially alter the nutrient uptake of the cell [87].

## 5. Evolution of Genome Content

### 5.1. Expansive Evolution

Similar to cellular genomes, giant virus genomes undergo both expansive and reductive genome evolution. The increased genome size in giant viruses can be attributed to gene duplications, de novo gene origination, and lateral gene transfers (LGTs, or horizontal gene transfers [HGTs]) from cellular organisms or other viruses [13,55,56,135,136,137]. Among these sources, LGTs generally bring in more innovative functions to viral genomes. Their identification can provide insights into virus-host interactions, connections between viruses and their current or past hosts, and how hosts play a role in shaping viral genomes. 

Laterally acquired genes in giant viruses largely fall into two categories: recently acquired genes from current hosts or related organisms [73,79,87,115] and anciently acquired, divergent viral homologs from an unknown source that sometimes form a clade with only viral and metagenomic sequences (e.g., type-1 rhodopsins [10,14]). It is notable that for some recent LGTs, viral genes demonstrate somewhat higher sequence divergence than their closest eukaryotic homologs in phylogenetic trees [73,79,87]. This might be due to generally higher evolutionary rates in giant viruses, though analyses of closely related marseilleviruses suggest they do not evolve faster than cells [138]. Alternatively, the transferred viral genes could be relieved from purifying selection, since host cells already have the same genes. This could allow viral homologs to acquire distinct functional properties that alter cellular behaviors upon infection [87]. Another interesting observation is the multiple independent acquisitions of the same gene across viral lineages with similar hosts, such as algae. Examples include potassium channels that have been repeatedly gained by viruses of marine and freshwater, unicellular and multicellular algae [51], and sodium/phosphate transporters with at least three independent events in coccolithovirus, *Ostreococcus* virus, and *Bathycoccus* virus, respectively [121].

### 5.2. Reductive Evolution

Giant viruses with larger genome size can potentially better manipulate specific hosts in a variety of pathways and cellular processes, but there are clearly factors that limit their genome size or cause reductive genome evolution as in other parasitic entities. Random gene losses intrinsically lead to genomic reduction during viral evolution [139]. There is almost no limit to the genes that can be lost, even genes central to information processing. Largely speaking, the repertoires of translation-related genes are the most variable [66], followed by transcription-related genes and then by genes for DNA replication [13,55,139]. A 16% reduction in genome size was observed in mimivirus subcultured 150 times in axenic *Acanthamoeba* cultures, which was accompanied by marked changes in virion morphology [140]. This illustrates how hosts and environment can cause fast genome size changes in giant viruses. Substantial genome size variation between closely related strains [56] and sister viral lineages (Figure 1) also point to highly dynamic expansive and reductive evolution at work.

Viral genomes and gene contents can also be shaped by certain host factors such as host genome size. It was shown that the burst size of phytoplankton dsDNA viruses correlates with host-to-virus genome size ratio [141]. Host genome size as a limiting factor can partially explain why, for example, prasinoviruses of Mamiellales, which have the smallest cell and genome size in Chlorophyta, have some of the smallest genomes in Phycodnaviridae or among all alga-infecting giant viruses [142]. Compared with its sister group chlorovirus (Figure 1), the prasinovirus genomes could have undergone reduction in size during evolution.

### 5.3. Generalist Viruses and Genome Evolution

The host range of a virus is determined by its genome, including the encoded genes and their regulation. On the other hand, a host can shape the genomes of its viruses, selecting for those better adapted to the host. This apparently chicken-or-egg relationship poses the question on how viruses can jump between distantly related eukaryotic host lineages like those of *Phycodnaviridae* and *Mimiviridae*. Here, we approach this question by proposing that there exist specialist viruses infecting only a specific host lineage and generalist viruses which can replicate in multiple eukaryotic host lineages across supergroups. After a generalist virus acquires genes that aid in the infection of a specific host, it can become a more specialized virus or remain as a generalist with more successful infection of the specific host. With higher replication success, more specialized viruses gradually become the dominant virus of the specific host, which could be why most viral lineages with isolated members are only known to infect a specific eukaryote lineage. True generalist viruses that can infect eukaryotes from different supergroups are unknown to date, either because they are less abundant and more difficult to isolate or because we have not explored the entire host range of the isolated giant viruses. There could be a pool of generalist viruses in the environment that would have been detected in metagenomic sequencing, where MAGs are the most abundant from *Mimiviridae* and *Phycodnaviridae* [17,81]—the two families with the most divergent eukaryotic hosts (Figure 1 and Section 3). In line with this generalist hypothesis for host range variation, the multiple independent acquisitions of similar membrane transport genes in the two families (Section 5.1) could correspond to transitions from generalist to specialist viruses infecting different algal or protist lineages. 

### 5.4. Origin of Giant Viruses and Their Families

An even more challenging question is what kind of hosts were infected by ancient NCLDVs, including the ancestors of NCLDVs and of each NCLDV family. Answers to this question would depend on our understanding of the genomic compositions of these ancient viruses. Although it can be still disputable [139], NCLDVs are generally believed to have evolved from a common ancestor. Phylogenomic and comparative genomic analyses suggest that the NCLDV common ancestor had a small viral genome [13] rather than that of a cellular organism [143], but what this common ancestor was like and its relationship to cellular eukaryotes are much more debated. Based on the phylogeny of two DDRP subunits, an NCLDV-early hypothesis was proposed where both NCLDVs and the individual NCLDV families originated before the last eukaryotic common ancestor (LECA), which is close to 2 billion years ago [144], and had infected a lineage of “proto-eukaryotes” that led to LECA [88]. Given the archaeal [145] and bacterial [146] ancestry of eukaryotic genomes, such “proto-eukaryotes” would be more like prokaryotes than at least LECA and its descendants. However, no NCLDV-like infection in prokaryotic cells has been reported so far. As single-gene trees can be misleading in inferring the ancient past of eukaryotes [147], it is also disputable whether we could take the virus-cell DDRP tree at face value [148] and assume they have evolved in viral lineages without being lost, regained, or replaced, which are especially problematic when inferring deep viral phylogenies [139]. Besides, if the NCLDV families *Iridoviridae*, *Marseilleviridae*, and *Pithoviridae* had originated before LECA [88], it is difficult to imagine that today they have such confined host range across the eukaryotic tree of life (Figure 1).

On the contrary, an NCLDV-late view would suggest a eukaryotic host for the NCLDV common ancestor. The deep divergence of shared genes is not necessarily the actual divergence among the giant viruses themselves, because they, as compartments of genes, can acquire divergent genes from different domains of cellular life. There is no doubt that widely-occurring core genes strongly shape the biology of giant viruses and are phylogenetically related. Nevertheless, this does not mean that they all have been passed on, together and vertically, through the deep bifurcations as depicted in their concatenated gene tree. Under the NCLDV-late hypothesis, the association between giant viruses and eukaryotes could have taken place later than the LECA origin and then spread across major eukaryotic lineages, as what could happen within just a single NCLDV family (*Mimiviridae* and *Phycodnaviridae*). In contrast to the obscure deep inter-familial relationships, NCLDVs form more coherent groups at the family level, which is true in terms of the number of shared genes and the viral biology [13,55] and probably especially so for families with similar genome size and lower sequence divergence. For families of viruses with similar hosts (*Marseilleviridae*, *Poxviridae*, and *Iridoviridae*), their ancient hosts were most likely from the same host lineage, but it would be more difficult to infer the ancestral host of *Phycodnaviridae* and of *Mimiviridae*. Even more challenging is to understand and predict genome variation across giant viruses. Rampant occurrence of LGTs is known to cause extensive gene content variation in prokaryotes, even among strains with highly similar core gene sequences [149,150,151], such that the 3% most vertically inherited genes are not predictive of the rest of the genome [152]. With limited evidence for long-term verticality plus substantial genome size differences, little can be inferred about giant virus gene contents from a phylogenetic tree based on a concatenated alignment of 10 genes that are not devoid of conflicting signals. More comprehensive whole-genome analyses are needed to determine the factors affecting gene content evolution both within and across giant virus families.

## 6. Future Perspective

Giant viruses demonstrate at least three unconventional features. Their bacterial-sized virions and genomes defy the idea that viruses are small infectious entities. Identification of new giant viruses from global ecosystems revealed the enormous diversity of their hosts across the now better resolved tree of eukaryotes. The functional potential of their genomes revolutionized our knowledge of how viruses can manipulate the host to complete their replication cycle. In a way, giant viruses function like powerful, innovative, yet often lethal, plug-ins in the program of eukaryotic life. This group of ubiquitous and ecologically important viruses will continue to be a source of exciting findings. In addition to crucial endeavors to isolate new strains, infection assays, metagenomics, single-particle genomics, functional characterization, and virus-host genomic analyses are expected to shed light on their biology, natural host range, virus-host interactions, and genome evolution within and across families.

## Figures and Tables

**Table 1:** A list of representative giant viruses and their morphological and genomic features.

Family	Virus (Lineage)	Shape	Size (nm)	Genome Size (kb)	DNA	tRNAs	RNA Polymerase Subunits [54]	Replication Cycle	Reference
*Pandoraviridae* (*Phycodnaviridae*)	*Pandoravirus salinus* (Pandoravirus)	Ovoid with a pore	1200 × 500	2770	Linear	3	4	Nuclear and cytoplasmic	[5,55,56]
*Phycodnaviridae*	*Paramecium bursaria Chlorella* virus 1 (Chlorovirus)	Icosahedral with a spike	170	331	Linear	11	0	Nuclear and cytoplasmic	[7,19]
*Mimiviridae*	*Acanthamoeba polyphaga* mimivirus (Mimivirus)	Icosahedral (with fibers)	390 (630)	1181	Linear	6	9	Cytoplasmic	[55,57]
*Pithoviridae*	*Pithovirus sibericum* (Pithovirus)	Ovoid with a capped pore	1500 × 800	610	Circular	0	4	Cytoplasmic	[6,32,55]
*Marseilleviridae*	*Marseillevirus marseillevirus* (Marseilleviridae A)	Icosahedral	250	368	Circular	0	3	Cytoplasmic, involving the nucleus	[29,58,59]
*Ascoviridae* (*Iridoviridae*)	*Spodoptera frugiperda* ascovirus 1a (Ascovirus)	Bacilliform or allantoid	400 × 130	157	Circular	0	4	Nuclear and cytoplasmic	[60,61]
*Iridoviridae*	Frog virus 3 (Alphairidovirinae)	Icosahedral	175	106	Linear	0	2	Nuclear and cytoplasmic	[18,62]
*Asfarviridae*	African swine fever virus BA71V (Asfarvirus)	Icosahedral	200	170	Linear	0	7	Nuclear and cytoplasmic	[63,64]
*Poxviridae*	Vaccinia virus (other Chordopoxvirinae)	Brick-shaped	310 × 240	195	Linear	0	9	Cytoplasmic	[65]

**Table 2:** A glimpse of the functional diversity of protein-encoding genes in giant viruses.

COG Category	Function	Distribution	Putative LGT Source	Reference
**Cellular Processes and Signaling**				
[M] Cell wall/membrane/envelope biogenesis	Hyaluronan synthesis	Chlorovirus	-	[89]
	Fucose synthesis	Chlorovirus	-	[90]
	L-rhamnose synthesis	Chlorovirus; Mimivirus	Trebouxiophyceae; eukaryotes	[91]
	3-deoxy-D-manno-octulosonate synthesis	Cafeteriavirus	Phagocytosed bacteria in the *Cafeteria* host	[9]
	4-Amino-4,6-dideoxy-D-glucose (Viosamine) synthesis	Mimivirus	-	[92]
[O] Posttranslational modification, protein turnover, chaperones	Protein glycosylase	Chlorovirus; Mimivirus	-	[93,94]
	Prolyl 4-hydroxylase	Chlorovirus	-	[95]
	Sulfhydryl oxidase	Asfarvirus; Mimivirus	-	[96,97]
	Isomerization of peptide bonds (Cyclophilin)	Mimivirus	-	[98]
**Information Storage and Processing**				
[J] Translation, ribosomal structure, and biogenesis	Aminoacyl tRNA synthetase	*Mimiviridae* except Mesomimivirinae; Pandoravirus; Orpheovirus	-	[13]
	Translation factors	*Mimiviridae*; Chlorovirus; *Pandoraviridae*; *Marseilleviridae*; *Asfarviridae*; Alphairidovirinae; *Pithoviridae*; Alphaentomopoxvirus	-	[13]
[K] Transcription	DNA-dependent RNA polymerase (DDRP) subunits	Most NCLDVs except many phycodnavirids	-	[37,54]
	Transcription elongation factors (TFIIS)	Most NCLDVs	-	[54]
	General transcription factors (TBP-like)	Some phycodnavirids and *Mesomimivirinae*	-	[54]
	General transcription factors (TFIIB-like)	Asfarviridae; *Mimiviridae*; *Marseilleviridae*; Pithovirus; Prasinovirus	-	[54]
[L] Replication, recombination, and repair	DNA Glycosylase	*Marseilleviridae*; most mimivirids; *Poxviridae*	-	[99]
	NAD/ATP dependent DNA ligase	Most NCLDV families	-	[100]
**Metabolism**				
[C] Energy production and conversion	Tricarboxylic acid (TCA) cycle	*Mimiviridae* MAGs; *Prymnesium* virus; Pandoravirus	-	[17,77,101]
	Cellular fermentation	*Tetraselmis* virus	*Tetraselmis* or related chlorophytes	[73]
[C] Energy production and conversion; [T] Signal transduction mechanisms	Rhodopsin	*Phaeocystis* virus; Choanovirus; metagenomic contigs	-	[10,14,79,102]
[E] Amino acid transport and metabolism	Polyamine synthesis	Chlorovirus	-	[103]
	Amino acid synthesis	Prasinovirus	Chlorophyta or bacteria	[25]
[F] Nucleotide transport and metabolism	Nucleoside-diphosphate kinase	Mimivirus	-	[104]
[G] Carbohydrate transport and metabolism	UDP-N-acetylglucosamine synthesis	Chlorovirus; *Mimiviridae*	Bacteria; -	[105,106]
	UDP-2-acetamido-2,6-dideoxy-hexose synthesis	Megavirus	-	[107]
	Cell wall (polysaccharide) degradation	Chlorovirus; Mimivirus	Non-Chloroplastida sources; -	[108,109,110,111,112,113]
[I] Lipid transport and metabolism	Sphingolipid synthesis	Coccolithovirus	Isochrysidales	[24,114,115]
[P] Inorganic ion transport and metabolism	Potassium ion channel	Chlorovirus; Phaeovirus; Prasinovirus; many *Mesomimivirinae* viruses	-	[51,116,117,118,119]
	Aquaporin	Chlorovirus	-	[120]
	Sodium/phosphate symporter	Coccolithovirus; Prasinovirus	Isochrysidales; Mamiellales	[121,122]
	Potassium ion transporter	Chlorovirus	Trebouxiophyceae	[121,123]
	Ammonium transporter	*Ostreococcus* virus; *Haptolina* virus	*Ostreococcus*; -	[87,121]
	Calcium-transporting ATPase	Chlorovirus	-	[124]
	Cu/Zn superoxide dismutase	Betaentomopoxvirus; Chlorovirus; Megavirus	-	[125,126,127]

## Supplementary Materials

The following are available online at https://www.mdpi.com/1999-4915/12/11/1337/s1. Table S1: Giant virus strains and their phylogenetic placement, genome information, and hosts.
